# Alginate Hydrogel Protects Encapsulated Hepatic HuH-7 Cells against Hepatitis C Virus and Other Viral Infections

**DOI:** 10.1371/journal.pone.0109969

**Published:** 2014-10-13

**Authors:** Nhu-Mai Tran, Murielle Dufresne, François Helle, Thomas Walter Hoffmann, Catherine François, Etienne Brochot, Patrick Paullier, Cécile Legallais, Gilles Duverlie, Sandrine Castelain

**Affiliations:** 1 UMR CNRS 7338 Biomechanics and Bioingineering, University of Technology, Compiègne, France; 2 EA4294 Department of Fundamental and Clinical Virology, University of Picardie Jules Verne, Amiens, France; UMR Inserm U1052/CNRS 5286, France

## Abstract

Cell microencapsulation in alginate hydrogel has shown interesting applications in regenerative medicine and the biomedical field through implantation of encapsulated tissue or for bioartificial organ development. Although alginate solution is known to have low antiviral activity, the same property regarding alginate gel has not yet been studied. The aim of this work is to investigate the potential protective effect of alginate encapsulation against hepatitis C virus (HCV) infection for a hepatic cell line (HuH-7) normally permissive to the virus. Our results showed that alginate hydrogel protects HuH-7 cells against HCV when the supernatant was loaded with HCV. In addition, alginate hydrogel blocked HCV particle release out of the beads when the HuH-7 cells were previously infected and encapsulated. There was evidence of interaction between the molecules of alginate hydrogel and HCV, which was dose- and incubation time-dependent. The protective efficiency of alginate hydrogel towards HCV infection was confirmed against a variety of viruses, whether or not they were enveloped. This promising interaction between an alginate matrix and viruses, whose chemical mechanisms are discussed, is of great interest for further medical therapeutic applications based on tissue engineering.

## Introduction

Among marine algae polysaccharide-based biomaterials, alginate is currently used in biomedical and pharmaceutical areas for wound dressing, as an ointment for burns, or as a formulation aid in controlled drug delivery systems [1]–[3]. Thanks to its biosafety and biocompatibility, alginate is also commonly used for tissue and cell immobilization by means of a bioencapsulation process [4]. Cells are entrapped within spherical alginate beads whose hydrogel structure protects them from mechanical stress while ensuring exchanges of nutrients or waste molecules within the surrounding medium. The immuno-isolation provided by alginate encapsulation is undoubtedly the major advantage of this technology when intended for transplantation or tissue regeneration. In the case of type I diabetes, twenty-five years of preclinical studies have recently made possible significant progress in the implantation of encapsulated Langerhans islets in patients [5]. Compared to other biopolymers, the considerable success of alginate used for microencapsulation relies upon the middle conditions required for the gelation process. Alginate salts, such as sodium-alginate (Na-alg), are composed of residues of *D*-mannuronic acid (M) and *L*-guluronic acid (G) covalently (1–4)-linked in homo- or hetero-blocks, and which have a high affinity for divalent or trivalent ions. Calcium ions interact through ionic crosslinking with the carboxylate groups of monosaccharide residues allowing the formation of a three dimensional (3D) network between polymeric chains, with limited effects on cell viability.

Numerous works described the antiviral activity of algal carbohydrate polymers [6]–[7] leading to promising therapeutic applications when used either alone or associated with existing antiviral drugs. These polysaccharides are extracted from the cell walls of red, brown or green algae from which they account for more than 50% of the dry weight. Besides their considerable structural diversity, all of these polymers are negatively charged and, in most cases, present a high sulfation level. Their antiviral activities target a broad spectrum of human pathogens including enveloped viruses such as human immunodeficiency virus (HIV) [8]–[9], herpes simplex virus (HSV) [10], human cytomegalovirus (HCMV), dengue virus [11], and non-enveloped viruses, such as hepatitis A virus (HAV) and human papillomavirus (HPV) [12]–[13]. Based on their safety and low toxicity, marine polysaccharides are interesting solutions for limiting viral infections in clinical contexts. Although experiences using marine polymers as an orally-delivered agent have been described [14], only a few clinical studies have been conducted so far [15]. The well-known anticoagulant activity of most sulfated polysaccharides, associated with their high molecular weight which is incompatible with free diffusion towards tissues, explains the obstacles to their use as natural compounds in *in vivo* conditions [14], [16]–[17]. Structural modifications by means of chemical or enzymatic processes can be requested to meet clinical constraints [18].

Although alginate antiviral activity is described as low compared to many other marine polysaccharide compounds, we hypothesized that this property could benefit cells entrapped in calcium-alginate (Ca-alg) beads for further use as implanted tissue or organ supply. For this purpose, using a simple extrusion process, we encapsulated human hepatoma-derived cells (HuH-7), a specific cell line which is up to now the most employed cellular model recognized for both its high permissiveness with regard to hepatitis C virus (HCV) infection and its ability to produce and secrete HCV particles [19]. The aim of this study was thus to investigate the potential protective effect of Ca-alg hydrogel encapsulating hepatic cells against HCV infection.

## Materials and Methods

### Alginate solution

Na-alg from *Macrocystis pyrifera* (brown algae), medium viscosity (Sigma-Aldrich) was used at 1.5% (w/v). Its molecular weight ranged from 80,000 to 120,000 Da, G/M = 33/67, viscosity at 2% = 2000 cP. To prepare the Na-alg solution, the alginate powder was dissolved within a sterile saline solution (154 mM NaCl solution buffered with 10 mM Hepes, pH 7.4). The mixture was subsequently filtered using a 0.2 µm membrane leading to a sterile Na-alg sol.

### 2D cell cultures

Hepatoma HuH-7 and transduced HuH-7-RFP-NLS-IPS cell lines were used. HuH-7 (RCB1366) cells were kindly provided by Jean Dubuisson (Institut de Biologie de Lille, France) and were plated in 25 cm^2^ flasks, cultured in Glutamax-supplemented DMEM with 10% fetal bovine serum. HuH-7-RFP-NLS-IPS cells were obtained by transduction of HuH-7 cells with lentiviral pseudo-particles expressing the RFP-NLS-IPS reporter. Briefly, lentivirus pseudo-particles were generated by co-transfection of 293T cells with TRIP-RFP-NLS-IPS (kindly provided by C.M. Rice, Rockefeller University, New York, USA), HIV gag-pol, and vesicular stomatitis virus envelope protein G (VSV-G) encoding plasmids as described previously [20]. HuH-7 cells were transduced by overnight incubation with lentivirus pseudo-particles at 37°C to obtain cell lines stably expressing the red fluorescent protein (RFP) on the outer membrane of the mitochondria. The translocation of the cleavage product RFP-NLS from the cytoplasm to the nucleus characterized the HCV infected cells. All cells were grown to 80-90% confluence in the same culture conditions before experiments to avoid any discrepancy in cell passage, cell density or medium quality.

### Encapsulated cell cultures

Encapsulated cell cultures were established using HuH-7 or HuH-7-RFP-NLS-IPS cells according to previously described techniques [21]. Cell density in the Na-alg sol was 500 000 cells/mL. Droplets of this mixture were obtained by a classical extrusion process with co-axial air flow. The parameters for the encapsulation process, including air and alginate flows, distance between the needle and the gelation bath surface, were experimentally determined to produce spherical droplets. The droplets formed were gelified in a CaCl_2_ bath (115 mM CaCl_2_, 154 mM NaCl buffered with 10 mM HEPES) for 15 min at room temperature, leading to ionically crosslinked alginate beads (Ca-alg beads) with an expected diameter of about 600 µm. Following the cell encapsulation stage, various volumes of beads were transferred to tissue culture 6-well plates with 1 mL or 2 mL of complete DMEM medium added on a 3D moving plate. The encapsulated cell cultures were maintained in a 5% CO_2_ incubator at 37°C.

Under certain conditions, the encapsulated HuH-7 cells were extracted from the beads after degelification based on alginate lyase treatment. Briefly, Ca-alg beads were washed twice with sterile PBS solution and incubated with alginate lyase solution (Sigma, 1 mg/mL) at v/v for 15 min at 37°C. The solution was then neutralized by addition of PBS and centrifuged. The cell sediment was recovered and plated in tissue culture 6-well plates with 2 mL of complete DMEM medium added.

### High-resolution cryo-Scanning Electron Microscopy (cryo-SEM)

High-resolution cryo-SEM (Hitachi S4500 field emission gun SEM equipped with a dedicated Polaron LT 7480 cryopreparation device, Orleans University) was used to investigate the 3D structure of the Ca-alg bead network in the absence of cells, as previously described [21]. Briefly, beads from the samples were cryofixed by plunge-freezing them in nitrogen slush at -210°C then fractured with a cold metal rod. The samples were transferred into the SEM chamber, where sublimation of the surface ice formed from the interstitial water of the sample was obtained by progressively increasing the temperature up to about −70°C. The temperature of the sample was then lowered back to about −110°C, making possible complete stabilization of the sample. The observations were performed at 1 kV without any sample coating.

### Epifluorescence microscopy

Cell viability within the beads was assessed qualitatively using fluorescence staining with propidium iodide and acridine orange (Sigma Aldrich, France) under a confocal microscope (DMI 6000B, Leica, France). A fluorescence microscope (Nikon Eclipse TE2000U) was used to characterize the infected HuH-7-RFP-NLS-IPS cells, by translocation of the RFP-NLS product from the cytoplasm to the nucleus. Nuclei were counterstained with DAPI.

### HCV infection

HuH-7 and HuH-7-RFP-NLS-IPS cells encapsulated in beads or in 2D cultures used as controls were in contact with one of the two following HCV particle types: (i) JFH1-CS-A4 (i24) virus (JFH1 virus), an adapted version of the full-length JFH1 virus (genotype 2a; GenBank access number AB237837; kindly provided by T. Wakita) which contains mutations leading to amino acid changes F172C and P173S at the C-terminus of the core protein [22]. Both mutations have been shown to increase the viral titers [23]; (ii) JFH1-CS-A4-RLuc-TM virus (JFH1 -RLuc virus) which contains a *Renilla* luciferase reporter gene as described previously [22].

Encapsulated and 2D cell cultures were incubated with the viral particles for 4h on 3D moving plates, the medium was then discarded and replaced with fresh medium. All experiments to assay HCV particles in the supernatant were performed 72h post-infection.

### HCV particle quantification

The HCV particles were detected using different methods to assess viral RNA replication (RNA quantification) or the particle infectivity of JFH1 and JFH1-RLuc by FFU titration and luciferase activity respectively.

- HCV RNA quantification: in this case, the quantification was performed directly in the supernatant of the encapsulated or 2D (control) cell cultures. Briefly, HCV RNA was extracted using the QiaAmp viral RNA mini kit (Qiagen). cDNAs were synthesized using a High Capacity cDNA Reverse Transcription kit and random hexamers as described by the manufacturer (Applied Biosystems). Amplifications were carried out with the TaqMan Universal PCR master Mix on an ABI 7900HT Sequence Detection System (Applied Biosystems) using primer and probe sets for HCV RNA [24].
- FFU titration: HCV infectious particle detection in the supernatants could be assessed by secondary infection of naive HuH-7-RFP-NLS-IPS cells, cultivated under 2D conditions. Briefly, 2D cell cultures were incubated for 4h with the bead culture supernatant, the medium was then discarded and replaced with fresh medium. Seventy-two hours post-infection, particle infectivity was determined by Focus Forming Unit (FFU) titration. The infected cells were fixed with paraformaldehyde (4%) and checked for fluorescence translocation in the nucleus using a confocal microscope.
- Luciferase activity: in this case, JFH1-CS-A4-RLuc-TM virus particles had to be used for the infection stage. HCV infectious particle detection could then be performed by quantifying luciferase activity in 2D naive HuH-7 cell cultures incubated with the bead culture supernatant. Two dimension cell cultures were incubated for 4h with the bead culture supernatant, the medium was then discarded and replaced with fresh medium. Seventy-two hours post-infection, the cells were lysed by addition of 100 µl of *Renilla* luciferase Assay Lysis Buffer (Promega). Luminescence was measured according to the manufacturer's instructions on a Centro XS3 LB960 luminometer (Berthold Technologies). Fifty microliters of cell lysate were used to determine luciferase activity. Results are expressed as relative light units (RLU) and are reported as the means ± S.D.

### Other virus infection

The infectivity of different viruses from the enveloped virus family such as Sindbis virus (50 nm in diameter), and herpes simplex virus type 1 (HSV-1) (140 nm) or the non-enveloped family such as Poliovirus type 1 (30 nm) was assessed on encapsulated HuH-7 cells in alginate beads. The cytopathic effect of these viruses on HuH-7 cells was observed after 48 h post-infection and the virus titration (Tissue culture infectious dose 50% or TCID_50_) was determined using microscopy imaging analysis as previously described [25].

### Data analysis

Data are expressed as mean ± standard error of mean value. All results are representative of 3 independent experiments. Statistical analysis was performed using the non-parametric Kruskal-Wallis test in order to compare the differences between the groups.

## Results

### HuH-7 cell encapsulation into Ca-alg beads

**Figure 1:**
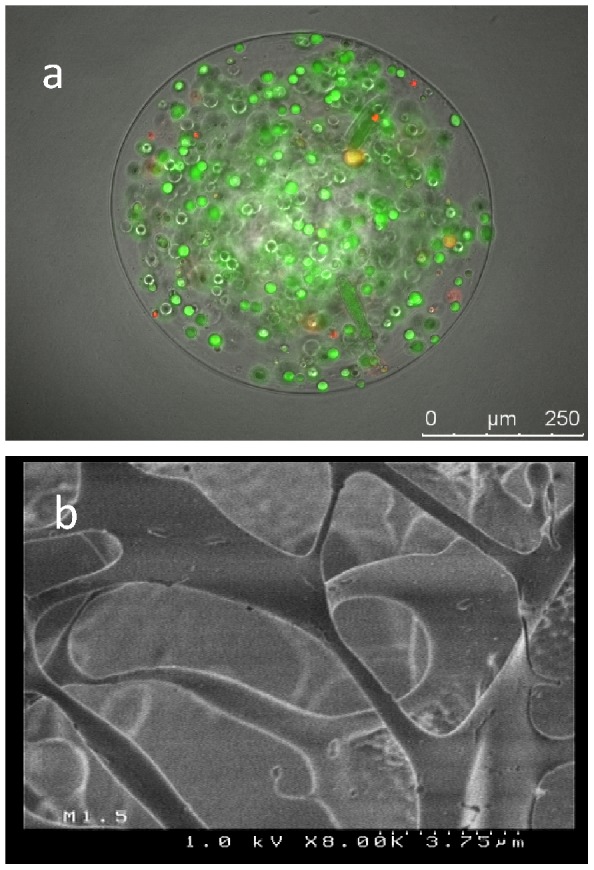
Characterization of HuH-7 cells encapsulated in Ca-alg beads at day 3 in culture. (A) Cell distribution in the Ca-alg beads. Cell viability was assessed using propidium iodide and acridine orange which stained dead and live cells, respectively. Scale bar 250 µm (B) The matrix porosity of Ca-alg beads was visualized by cryoscanning electron microscopy. Scale bar 3.75 µm.

The 3D cell culture was established by encapsulating HuH-7 cells at 500 000 cells/mL of Na-alg as described in the materials and methods section. The isolated cells were found to be homogeneously distributed into beads and maintained high viability post-encapsulation (Fig. 1A). The beads' mean diameter was about 600 µm. The beads showed a porous inner structure with a pore size estimated at 9.5+/−0.34 µm using cryo-SEM (Fig. 1B).

### Absence of infection by HCV when HuH-7-RFP-NLS-IPS cells were encapsulated in Ca-alg beads

**Figure 2:**
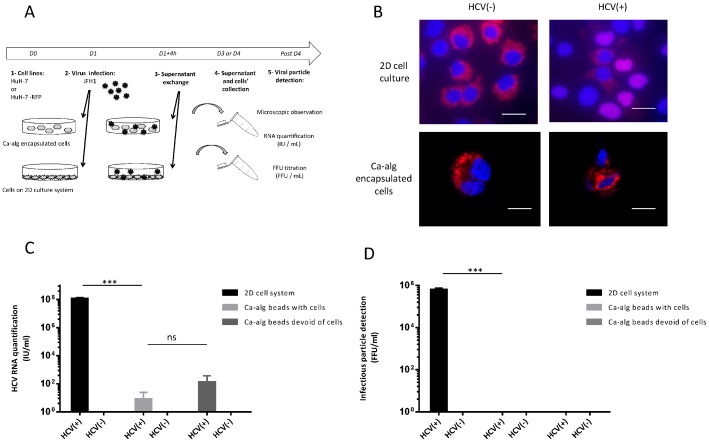
Absence of HCV infection by Ca-alg encapsulated cells. (A) Encapsulated cell cultures were established by encapsulating HuH-7-RFP-NLS-IPS cells within Ca-alg beads (500 000 cells/mL). Following the cell encapsulation stage, 400 µL of beads were transferred to tissue culture 6-well plates with 1 mL of DMEM medium added on 3D moving plates. After 4 h of contact with the JFH1 virus (HCV+) or without (HCV), the medium was removed and replaced with 2 mL of fresh complete DMEM medium. Ca-alg beads devoid of HuH-7 cells were used as controls. (B) Foci of infected cells (in 2D or in beads), identified by translocation of the cleavage product RFP-NLS from cytoplasm to nucleus, were visualized at 48 h by fluorescence microscope. Images are representative of three independent experiments. Nuclei were stained by DAPI. (C) The amount of HCV RNA was quantified in the bead supernatants by RT-qPCR. Results are expressed as HCV RNA IU/mL and are reported as the mean ± S.D. of triplicate measurements. (D) Viral titers were determined in the bead supernatants by FFU assay. Results are expressed as FFU/mL and are reported as the mean ± S.D. of three independent experiments. ***P<0.0001, ns: no significant difference.

In these experiments, HuH-7-RFP-NLS-IPS cells were used to characterize the infected cells (Fig. 2A). 48 h after a 4 h incubation period between HCV and Ca-alg bead hosting cells, microscopic visualization of the infected cell foci was performed. Translocation of a cleavage product from the cytoplasm to the nucleus was observed in the control 2D cell cultures, characterized by an overlay of DAPI and RFP fluorescent dyes. Interestingly, an absence of foci in the HuH-7-RFP-NLS-IPS cells was observed in the encapsulated cell system incubated with HCV, the red fluorescence remaining in the cytoplasm (Fig. 2B). To confirm the absence of new HCV cell culture (HCVcc) production in the supernatant of encapsulated HuH-7 cells, after 4 h of JFH1 virus incubation in contact with Ca-alg beads immobilizing HuH-7-RFP-NLS-IPS cells, the medium was removed and replaced by fresh complete DMEM medium. The production of new particles was assessed 72 h post-infection by measuring in the supernatant the amount of HCV RNA expressed as HCV RNA IU/mL (Fig. 2C). The HCV RNA was quantified at very low levels in the supernatant of the Ca-alg encapsulated cells, with no significant difference between the empty Ca-alg beads condition, i.e. beads devoid of cell, unlike the 2D cell cultures used as positive controls, which showed significant differences up to a 6 log fold change between both system cultures (*P*<0.0001). In the same way, no production of new HCVcc particles by FFU titration was measured compared to the 2D control cultures (Fig. 2D). This means that HuH-7 cells encapsulated in Ca-alg beads were not infected by the HCV added to the supernatant.

### Absence of HCVcc new particle production of previously infected and Ca-alg encapsulated HuH-7 cells

**Figure 3:**
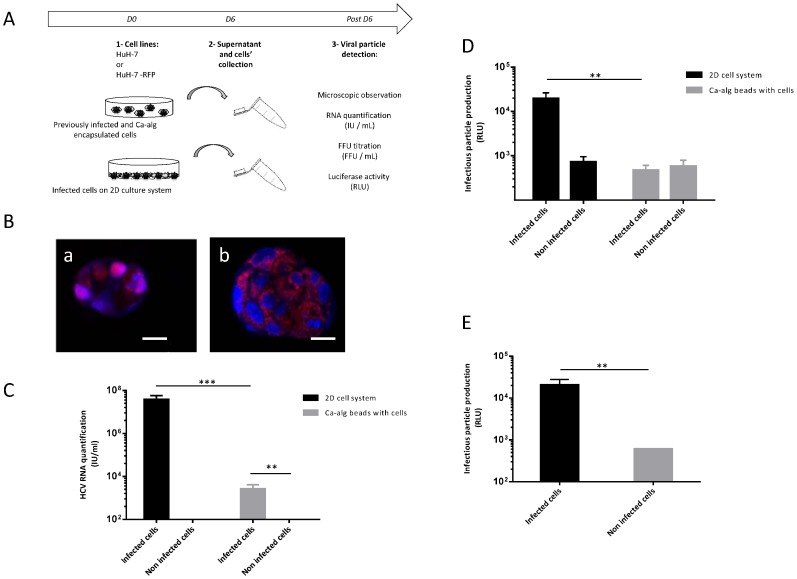
Absence of production of new HCVcc particles by previously infected and encapsulated cells. (A) Encapsulated cell cultures were established using JFH1-RLuc virus-infected HuH-7 cells or non-infected cells within Ca-alg beads. Following the cell encapsulation stage, 400 µL of beads were transferred in tissue culture 6-well plates with 1 mL of complete DMEM medium added on 3D moving plates. (B) Foci of infected (a) or non-infected (b) HuH-7-RFP-NLS-IPS cells identified by translocation of the cleavage product RFP-NLS from cytoplasm to nucleus, were visualized at 6 days post-encapsulation by fluorescence microscope. Images are representative of three independent experiments. Nuclei were stained by DAPI. Scale bar 20 µm. The supernatants of the bead culture cells were collected at day 6 and incubated for 4 h with HuH-7 cell 2D cultures. (C) The amount of HCV RNA was quantified in the bead culture supernatants by RT-qPCR. Results are expressed as HCV RNA IU/mL and are reported as the mean ± S.D. of triplicate measurements. (D) Infectious particle production was assessed luciferase assay on infected cells at 72 h post-infection. Results are expressed as RLU and are reported as the means ± S.D. of three independent experiments. (E) Infectious particle production was also assessed by measuring *Renilla* luciferase activities after bead degelification to free previously infected cells and plating them. Results are expressed as relative light units (RLU) and are reported as the means ± S.D. of three independent experiments. **P<0.001, ***P<0.0001.

To explore this phenomenon, cells were chronically infected with the JFH1-RLuc virus before their encapsulation in Ca-alg beads (Fig. 3A). After 6 days' culture on 3D moving plates, HuH7-RFP-NLS-IPS encapsulated cells were observed with fluorescence microscopy. The translocation of the RFP product from cytoplasm to nucleus showed that previously infected cells were able to achieve HCV replication when encapsulated in Ca-alg beads (Fig. 3B).

The quantification of HCV RNA was assessed by RT-qPCR (Fig. 3C) and by new HCVcc production in the culture supernatant by measuring luciferase activity 72 h post-infection (Fig. 3D). As expected, in the 2D system used as the control with an equivalent number of cells and encapsulated cells, the HCV RNA in the supernatant was quantified. Conversely, HCV RNA detected in the supernatant of infected and encapsulated cells was a 4 log fold change lower than in the 2D system supernatant (*P*<0.0001) but superior to the supernatant of the beads hosting non-infected cells. To determine the origin of the RNA detected in the culture medium of the encapsulated infected cells, the production of new HCVcc particles was quantified. In previously infected and encapsulated cell cultures, luciferase activity levels were under the threshold corresponding to non-infected and encapsulated cells. This suggested that no new HCVcc particles were released in the supernatant of the Ca-alg encapsulated infected cells. The viral RNA detected may then correlate with defective or degraded RNA from lysed infected cells in the beads.

In spite of their ability to achieve HCV replication, the stress inherent to the encapsulation process may affect the previously infected cells, as the infected cells are more sensitive than the non-infected ones, and influence viral production. To alleviate this hypothesis, Ca-alg beads were degelified by alginate lyase treatment and the cells recovered were plated on the 2D system. HCVcc particle production in the culture supernatant was assessed by measuring luciferase activity 72 h post-infection. As shown in Figure 3E, the capacity for HCVcc particle production in the chronically infected cells was not altered by the encapsulation and degelification processes. The restoration of HCVcc production in infected HuH-7 cells after bead degelification indicated that the absence of detection of HCVcc particles in the supernatant of Ca-alg encapsulated cells cannot be attributed to cell dysfunctions.

### Protective property of Ca-alg beads against HCV infection depends on the Ca-alg bead/virus volume ratio and time of incubation between HCV and Ca-alg beads

**Figure 4:**
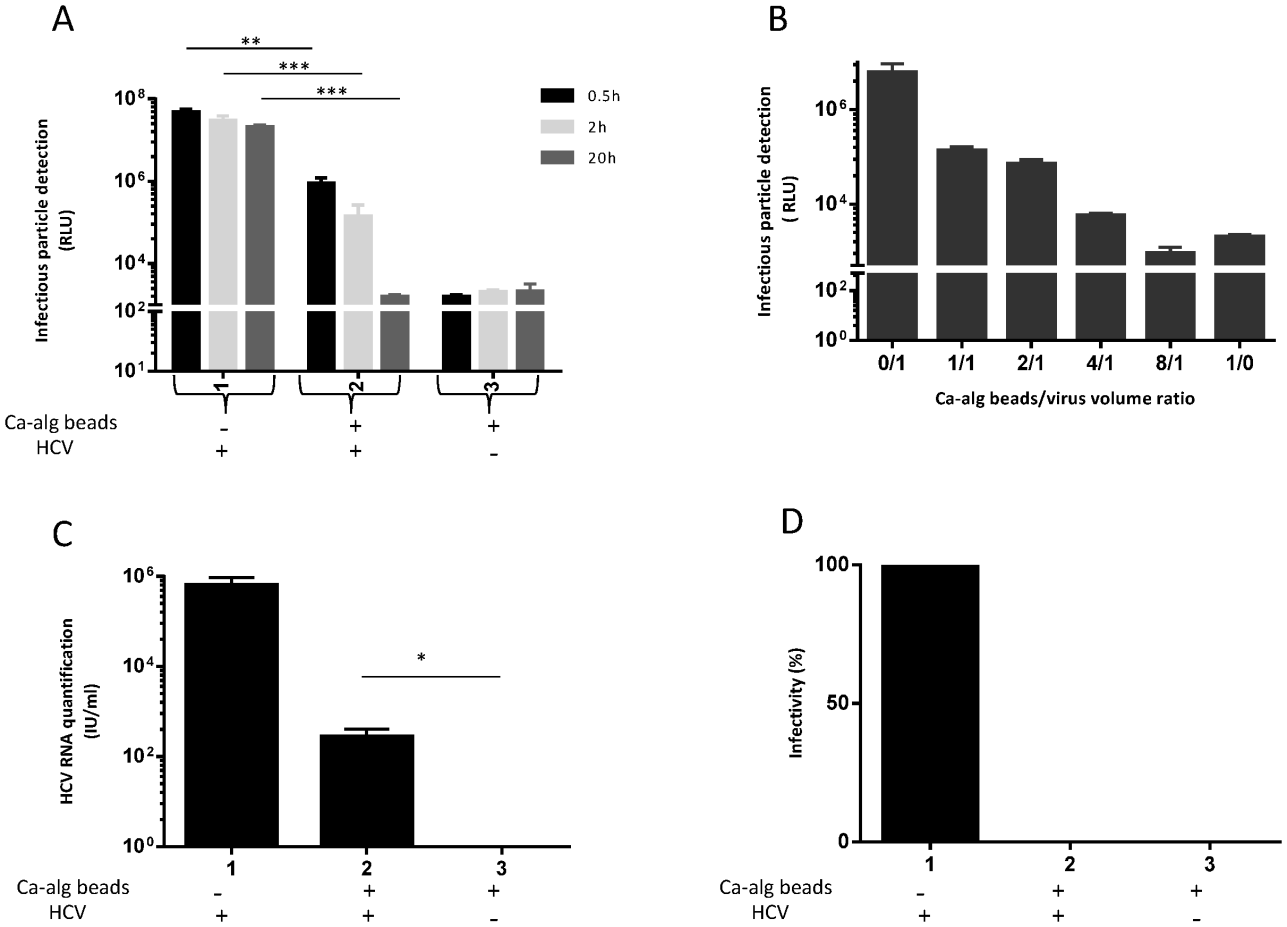
Protective property of Ca-alg beads against HCV infection is dependent on bead/virus volume ratio concentration and time of incubation. Ca-alg beads devoid of cells were produced. (A) 1200 µL of Ca-alg beads were transferred in tissue culture 6-well plates with 1 mL of complete DMEM medium added on 3D moving plates. After three incubation times (0.5, 2 and 20 h) at room temperature of JHF1-RLuc virus with a Ca-alg bead/virus volume ratio of 4/1, the supernatant was recovered and incubated for 4 h with HuH-7 cell 2D cultures. The detection of infectious particles was assessed by luciferase assay on infected cells at 72 h post-infection. Results are expressed as RLU and are reported as the means ± S.D. of three independent experiments. (B) The same experiment was performed with different Ca-alg bead/virus volume ratios (0/1 to 8/1) for 20 h. As previously described, the supernatant was recovered and incubated for 4 h with HuH-7 cell 2D cultures. Luciferase assays were performed on the infected cells at 72 h post-infection. The Ca-alg beads without the JFH1-RLuc virus were used as controls (Ctrl). Results are expressed as RLU and are reported as the means ± S.D. of three independent experiments. (C) After 20 h post-incubation of JFH1 virus in a Ca-alg bead/virus volume ratio of 4/1, the Ca-alg beads were washed, degelified by lyase treatment. Viral titers of the supernatants were determined by FFU assay. Results are converted into a percentage of infectivity. (D) Under the same incubation conditions as C), the amount of HCV RNA was also quantified in the supernatants (condition without beads) and from the Ca-alg bead products after lyase treatment (condition with beads) by RT-qPCR. Results are expressed as HCV RNA IU/mL and are reported as the mean ± S.D. of triplicate measurements. *P<0.05, **P<0.001, ***P<0.0001.

Taken together, the previous results suggested that the Ca-alg matrix may interact with HCV particles during the exchanges between the inner and outer parts of the beads. To challenge this assumption, various incubation conditions for Ca-alg beads devoid of cells with the JFH1-RLuc virus were analyzed on a 3D moving support. Firstly, after three incubation times (0.5, 2 and 20 h), the supernatant was collected and submitted to HCV particle detection by measuring luciferase activity. As shown in Fig. 4A, the number of HCV particles decreased in the supernatant after contact with the Ca-alg beads, and this decrease was directly linked to the time of contact between the virus and beads. The longer the contact time, the lower the number of infectious HCV particles in the supernatant, reaching the level of the condition of the Ca-alg beads without HCV. Secondly, different Ca-alg bead/virus volume ratios were tested (0/1 to 8/1). As could be expected, the interaction between the Ca-alg beads and virus was significantly linked to the bead/virus volume ratio. The absence of detection of HCV particles in the supernatant was observed when a bead/virus volume ratio of 8/1 (Fig. 4B) was attained. All these results suggest that the protective property of the Ca-alg beads against HCV was associated with trapping the viral particles by the Ca-alg hydrogel, which depended on the available and/or accessible surface of the materials in contact with them. Thus, the beads could retain HCV by a confinement of the particles in hydrogel porosity or by chemical interactions between the alginate molecules and viral particles.

### Protective property of Ca-alg beads against HCV depends on viral particle binding to Ca-alg beads

To determine whether the viral entrapment depended on physical or chemical interactions with the Ca-alg hydrogel, beads devoid of cells were incubated for 20 h with HCV, washed, and enzymatically digested by means of alginate lyase treatment. The alginate lyase is known to cleave the glycosidic bonds, freeing the monomers or oligomers from the alginate molecules [26]. The presence of HCV RNA detected in bead digestion products after alginate lyase treatment (Fig. 4C) associated with the absence of infectious particles (Fig. 4D) suggested that stable interactions link HCV envelope components to the Ca-alg matrix.

### Protective activity of Ca-alg beads against other enveloped and non-enveloped viruses

**Figure 5:**
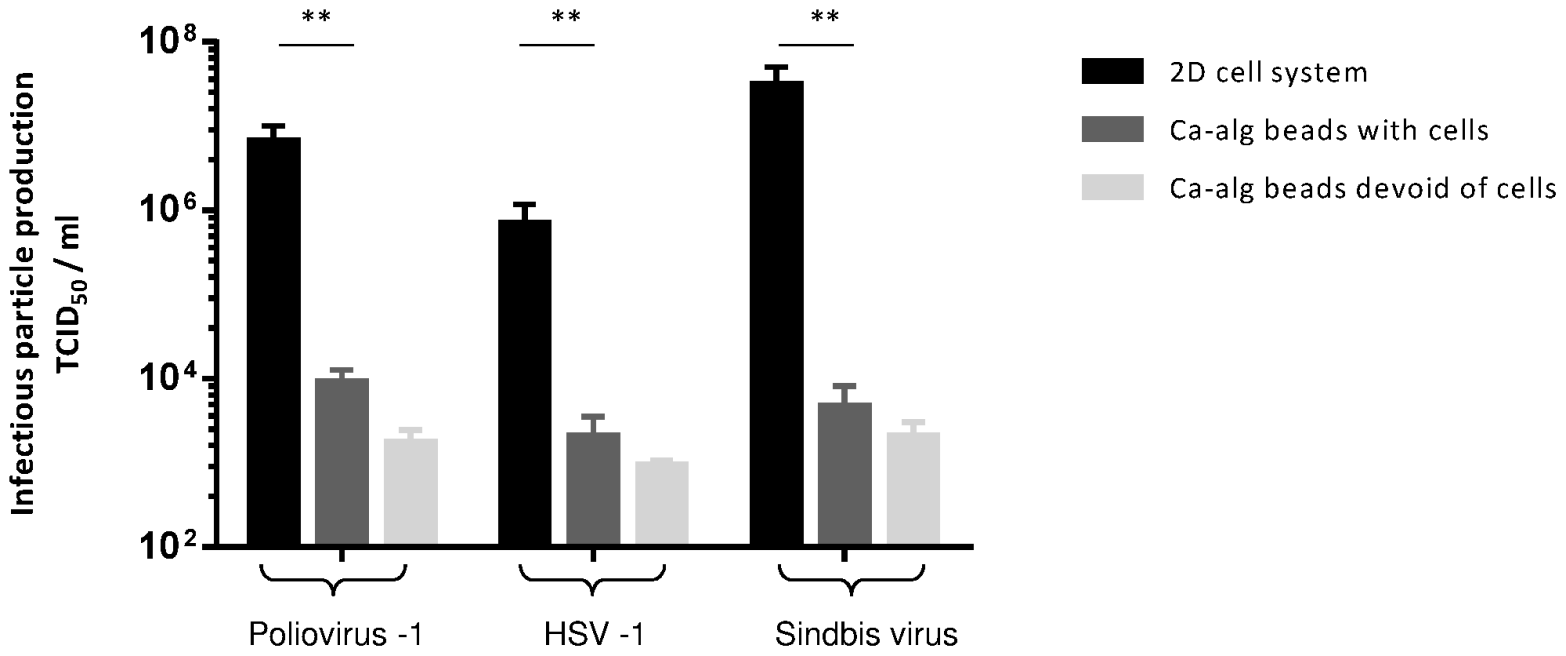
Protective property of Ca-alg beads against various enveloped and non-enveloped viruses. Following the HuH-7 encapsulation stage, 600 µL of beads were transferred to tissue culture 6-well plates with 1 mL of complete DMEM medium added on 3D moving plates. After 4 h of Sindbis virus, HSV-1 and Poliovirus type 1 infection, the medium was removed and replaced with fresh complete DMEM medium. The Ca-alg beads devoid of cells were used as controls. The supernatants of the cultured cells were recovered at 48 h and incubated for 4 h with HuH-7 cell 2D cultures. Infectious particle production was assessed by TCID_50_ titration using microscopy imaging analysis. **P<0.001.

To further characterize whether this Ca-alg gel property is specific to HCVcc particles or not, three other viruses were studied in similar Ca-alg encapsulation culture conditions. One non-enveloped virus (Poliovirus type 1) and two enveloped viruses (HSV-1 and Sindbis virus) were incubated for 4 h with 600 µL of beads encapsulating HuH-7 cells. Then, the supernatant was removed and replaced with fresh complete medium. After 48 h of incubation, the supernatants were used to infect HuH-7 cell 2D cultures at different dilutions. After 24 h, particle infectivity was determined by TCID_50_ titration. As shown in Fig. 5, a drastic decrease in the infectious titer (more than 2 fold for HSV-1 and 3 fold for poliovirus and Sindbis virus) was observed between supernatants harvested in 2D and encapsulated culture systems, respectively. These results were similar to those observed for the HCV infectivity tests. Altogether, these data suggest that the Ca-alg matrix activity was not specific to one virus but that this gelified polymer may interact indifferently with enveloped and non-enveloped viruses.

## Discussion

The present study showed for the first time a protective effect of alginate gel used as a matrix for HuH-7 cell microencapsulation against various pathogen viruses. To test the protective effect of Ca-alg beads against HCV infection, we encapsulated either HuH-7-RFP-NLS-IPS cells before submitting this cell system to HCV or previously infected cells. In all cases, no infectious HCVcc particle was produced in the culture medium. More precisely, the non-translocation of the cleavage products RFP-NLS from cytoplasm to the nucleus showed the inability of the HCV to access the entrapped cells or to activate the HCV receptors to enter into cells. However, our previous data suggested HuH-7 cell cultures in Ca-alg beads were a relevant model for HCV infection for two reasons: i) after their encapsulation in Ca-alg beads with optimized alginate composition, isolated HuH-7 cells proliferated and reorganized into multicellular aggregates [27]. The recovery of a differentiated state was confirmed by the polarized structure of the cellular aggregates, characterized by specific localization of tight junctions and polarity markers, providing evidence of the beneficial effect of the 3D environment culture in the Ca-alg matrix to reproduce a hepatic-like tissue. HuH-7 cells expressed specific receptors to HCV, such as receptors SR-BI and CD81, and co-receptors, claudin-1 and occludin, mimicking the *in vivo* configuration. HuH-7 cells embedded in Ca-alg beads were thus expected to recognize and interact with HCV on their plasma membrane, as was confirmed by recent studies when aggregated HuH-7 cells were cultivated without any matrix or embedded in Matrigel or in galactosylated cellulosic sponge [28]–[30]. ii) The second reason dealt with the structure of the Ca-alg matrix. In a previous work, various concentrations and viscosities of Na-alg solution were tested to produce beads with a porosity allowing the diffusion of VHC size particles. According to the high-resolution cryo-SEM analysis of the Ca-alg matrix and to dynamic diffusion test results, the Na-alg solution at 1.5% with medium viscosity was retained. In particular, the beads produced by this way were permeable to polystyrene nanoparticles up to 100 nm in diameter [27], which is compatible with the diameter of HCV (50 to 80 nm) [31]. Therefore, neither the biological properties of the HuH-7 cells nor Ca-alg bead porosity could explain the absence of viral production in the supernatant, when naïve or previously infected HuH-7 cells were encapsulated.

The barrier exerted by the Ca-alg network is composed of homo- and hetero-polymers of M and G residues whose negative charges of carboxylate groups interact with calcium to crosslink the polymer chains. The Ca-alg hydrogel maintains high negative charges, as it was analyzed by the potential zeta of beads [32]–[33]. The viral inhibitory effects due to ionic interactions are well documented, particularly among the sulfated polysaccharides derived from natural sources [16]. Studies on the structure-activity relationships of seaweed polysaccharides have underlined distinct molecular mechanisms for antiviral actions which inhibit different stages in the virion life cycle or prior to cell infection, *i.e*. by inactivating viruses before host cell contact [7]. Our results suggest that the high negative charges of Ca-alg hydrogel may interact with envelope components of HCV, blocking the viral particles in the gel environment or hindering specific interactions between viral compounds and specific membrane receptors. Similar results were obtained with low and medium viscosity alginate (data not shown) supporting the idea that polysaccharide chain length was not involved in the antiviral activity. These data were confirmed using pure alginate of medical grade quality (data not shown), meaning that no overshadowing responses associated to contaminations such as endotoxins were implicated in the viro-protective effect of Ca-alg gel. In addition, the results of the incubation time and dose-effect experiments, using different ratios of empty bead volume-to-viral charge, were in favor of an adsorption mechanism of the viral particles on the Ca-alg matrix.

Nevertheless, alginate molecules in suspension revealed low antiviral activity compared to other marine polysaccharides [16]. The chemical composition of M and G residues, which are naturally devoid of sulfated groups, explains the weak antiviral potency of alginate [34]. As an example, anti-HSV-1 activity of Na-alg was lower than that of sulfated polysaccharides, characterized by 50% inhibitory concentration values (IC_50_) in a range of 10 to 15 µg/mL, *i.e*. ten times higher than the IC_50_ of fucoidans, sulfated molecules [10], [34]. Cermelli *et al*. demonstrated antiviral activity of hyaluronic acid, a non-sulfated negatively-charged glycosaminoglycan, characterized by variable efficiency depending on the type of virus, at effective concentrations ranging from 1 to 4 mg/mL [35]. In the present work, beads were extruded from an Na-alg solution at 15 mg/mL, which is 1000 times greater than that of the alginate solution concentrations classically used for antiviral activity experiments [10], [33]. This high alginate concentration in hydrogel, generally varying from 0.5 to 2%, was reinforced by shrinkage of the beads during their immersion in the calcium solution used for the gelification stage [36], [37]. The negative charges concentrated in a limited volume and the spatial conformation of the polymers in Ca-alg gels might also support efficient antiviral activity [10]. Finally, our results support a non-specific Ca-alg-virus interaction regarding the efficiency of the Ca-alg viro-protective effect on a variety of viruses, whether enveloped or not. Cermelli *et al*. speculated on a structure-activity relationship of negatively-charged glycosaminoglycan involving general/non specific host cell-virus interactions [35]. Structure and sequence-based statistical analyses have demonstrated that positively-charged basic amino acids on viral proteins participate in binding to glycosaminoglycan receptors [38]. Ca-alg hydrogel may inhibit different viruses by interfering with the viral adsorption process via receptor entry blocking [7].

The recent progress made in bioengineered products provides a hopeful strategy for liver supply, offering a promising alternative to whole liver transplantation which suffers from an allogenic organ shortage crisis [39]. The allo- or xenotransplantation of hepatocytes encapsulated in alginate beads is an attractive approach to support host liver recovery and whose feasibility has been demonstrated in various animal models [40]–[41]. Mei *et al*. documented the beneficial influence of implantation of porcine encapsulated cells on survival rate and metabolic performances compared to free hepatocyte transplantation in a mouse model of liver failure [41], which was confirmed by the co-encapsulation of stem cells and hepatocytes [42]–[44]. Nevertheless, the use of allogenic or xenogenic cell microencapsulation for regenerative medicine is associated with certain risks in terms of virus-mediated infectious diseases provided from either the grafts or the recipients [45], which may ultimately have an impact on human health recovery. Given the numerous applications for microencapsulation in Ca-alg beads using a natural biomaterial approved by the U.S. Food and Drug Administration, the promising *in vitro* protective effect against viruses reported here is an innovative and attractive property of alginate gel with two new interesting advantages: first, viral infection by a retrovirus, an endogenous virus or a potentially unknown virus from the encapsulated cells cannot be transmitted to the patient, and, conversely, encapsulated cell functions cannot be hampered by a viral infection in the host. Numerous applications in the field of regenerative medicine may be concerned, such as cartilage repair, bone regeneration [46] or diabetes treatment by means of a bioartificial pancreas [47]. More generally speaking, the protective property of alginate gel against viruses may have applications extending far beyond biomedicine [48].

## Conclusion

Alginate hydrogel used as a matrix for HuH-7 cell microencapsulation has a protective effect against JFH1 HCV, Sindbis virus, HSV-1, and Poliovirus type 1 infection when these viruses were added to the supernatant. In addition, Ca-alg hydrogel blocked the release of HCV particles out of the beads when HuH-7 cells were previously infected and encapsulated. The use of Ca-alg beads devoid of cells in inhibitory experiments showed that the protective activity was dose- and incubation time-dependent, and depended on chemical interactions between the Ca-alg gel and HCV particles. Broadening this protective effect may have appealing applications in regenerative medicine.
